# Role of Ca^2+^ in the Control of H_2_O_2_-Modulated Phosphorylation Pathways Leading to eNOS Activation in Cardiac Myocytes

**DOI:** 10.1371/journal.pone.0044627

**Published:** 2012-09-06

**Authors:** Juliano L. Sartoretto, Hermann Kalwa, Takashi Shiroto, Simone M. Sartoretto, Michael D. Pluth, Stephen J. Lippard, Thomas Michel

**Affiliations:** 1 Cardiovascular Division, Department of Medicine, Brigham and Women's Hospital, Harvard Medical School, Boston, Massachusetts, United States of America; 2 Department of Chemistry, Massachusetts Institute of Technology, Cambridge, Massachusetts, United States of America; University of Sassari, Italy

## Abstract

Nitric oxide (NO) and hydrogen peroxide (H_2_O_2_) play key roles in physiological and pathological responses in cardiac myocytes. The mechanisms whereby H_2_O_2_–modulated phosphorylation pathways regulate the endothelial isoform of nitric oxide synthase (eNOS) in these cells are incompletely understood. We show here that H_2_O_2_ treatment of adult mouse cardiac myocytes leads to increases in intracellular Ca^2+^ ([Ca^2+^]_i_), and document that activity of the L-type Ca^2+^ channel is necessary for the H_2_O_2_-promoted increase in sarcomere shortening and of [Ca^2+^]_i_. Using the chemical NO sensor Cu_2_(FL2E), we discovered that the H_2_O_2_-promoted increase in cardiac myocyte NO synthesis requires activation of the L-type Ca^2+^ channel, as well as phosphorylation of the AMP-activated protein kinase (AMPK), and mitogen-activated protein kinase kinase 1/2 (MEK1/2). Moreover, H_2_O_2_-stimulated phosphorylations of eNOS, AMPK, MEK1/2, and ERK1/2 all depend on both an increase in [Ca^2+^]_i_ as well as the activation of protein kinase C (PKC). We also found that H_2_O_2_-promoted cardiac myocyte eNOS translocation from peripheral membranes to internal sites is abrogated by the L-type Ca^2+^ channel blocker nifedipine. We have previously shown that kinase Akt is also involved in H_2_O_2_-promoted eNOS phosphorylation. Here we present evidence documenting that H_2_O_2_-promoted Akt phosphorylation is dependent on activation of the L-type Ca^2+^ channel, but is independent of PKC. These studies establish key roles for Ca^2+^- and PKC-dependent signaling pathways in the modulation of cardiac myocyte eNOS activation by H_2_O_2_.

## Introduction

The endothelial isoform of nitric oxide synthase (eNOS) is robustly expressed in cardiac myocytes, and nitric oxide (NO) has been shown to play key roles in modulating cardiac function [1], [2], [3]. eNOS is a Ca^2+^/calmodulin-dependent enzyme that undergoes phosphorylation on multiple residues in response to extracellular stimuli, involving several protein kinases and phosphoprotein phosphatases. We have recently shown that hydrogen peroxide (H_2_O_2_) is a critical intracellular mediator that modulates eNOS phosphorylation and enzyme activation in cardiac myocytes [2]. However, the role of H_2_O_2_ in modulation of cardiac myocyte Ca^2+^ metabolism is less well understood, and there are major gaps in our understanding of the pathways connecting H_2_O_2_–dependent phosphorylation pathways, intracellular Ca^2+^ signaling, and eNOS activation.

Cardiac myocytes contain an astonishingly broad array of protein kinases, several of which may be modulated by H_2_O_2_. Some protein kinase C (PKC) isoforms are activated by H_2_O_2_, yet little is known about the modulation of eNOS by PKC in the heart. Other protein kinases expressed in cardiac myocytes that have been implicated in eNOS regulation include ERK1/2, MEK1/2, kinase Akt, AMPK, and the cyclic AMP-dependent protein kinase (PKA). Since abnormalities in PKC-modulated signaling pathways and alterations in intracellular Ca^2+^ metabolism have been implicated in cardiomyopathy and heart failure [4], [5], [6], we decided to explore the role of H_2_O_2_ in control of PKC activation, intracellular Ca^2+^ pathways, and eNOS phosphorylation responses in cardiac myocytes. Here we provide data that establish roles for Ca^2+^, PKC and PKA in modulating eNOS phosphorylation in response to H_2_O_2_, and identify the key protein kinase pathways that modulate H_2_O_2_–dependent NO synthesis in cardiac myocytes.

## Results

**Figure 1:**
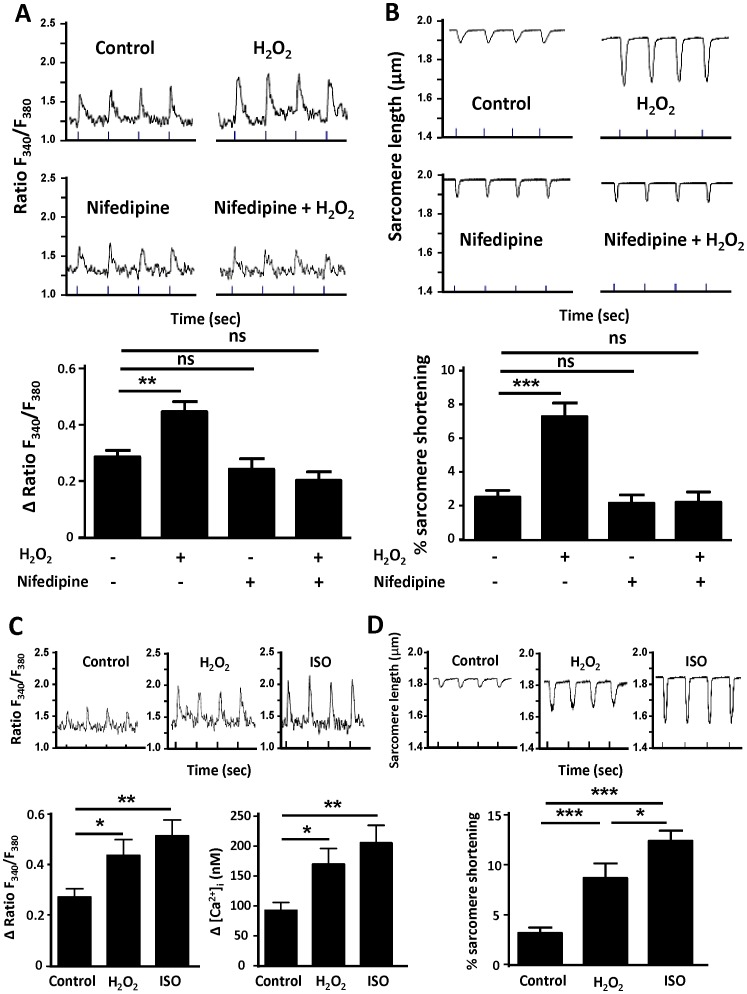
H_2_O_2_ treatment increases Fura-2 fluorescence and cardiac myocyte contractility. Panel A shows the effects of hydrogen peroxide (H_2_O_2_, 25 µM) on F_340_/F_380_ ratio in Fura-2 loaded adult mouse cardiac myocytes. Cells were loaded with Fura-2 AM (1 µM) for 20 minutes prior to microscopic analysis. Intracellular Fura-2 fluorescence was measured using electrically stimulated preparations (1 Hz, 5–10 volts). Representative tracings of Fura-2 ratio of cells treated with H_2_O_2_ or H_2_O_2_ in the presence of nifedipine are shown above, and pooled data are shown below measuring the Δ Fura-2 ratio in which peak height is subtracted from basal; between 9 and 23 cells were analyzed under each condition. Panel B shows representative sarcomere length traces of cardiac myocytes treated with hydrogen peroxide (H_2_O_2_, 25 µM) in the presence or absence of nifedipine. Below is shown pooled data analyzing contractility as deflections from the baseline sarcomere shortening, which was measured as the percentage of the baseline resting cell length following treatments as shown. Recordings were performed at room temperature and myocytes were stimulated at 1 Hz, 5–10 volts. The results of pooled data were analyzed from three independent experiments involving 11–30 cells each that yielded equivalent results. Panel C shows representative tracings of Fura-2 in cells treated with hydrogen peroxide (H_2_O_2_, 25 µM) or isoproterenol (ISO 0.1 µM) on F_340_/F_380_ ratio (upper panel); pooled data below show the Δ Fura-2 ratio (left panel), and intracellular calcium concentrations (right panel) between 9 and 19 cells are analyzed under each condition. Panel D shows representative sarcomere length traces of cardiac myocyte treated with hydrogen peroxide (H_2_O_2_, 25 µM) or isoproterenol (ISO 0.1 µM). Results of pooled data are below the representative tracings, and show the effects of hydrogen peroxide (H_2_O_2_, 25 µM) or isoproterenol (ISO 0.1 µM) on sarcomere length and percentage of sarcomere shortening; between 9 and 22 cells were analyzed under each condition. *indicates p<0.05; **indicates p<0.01; and ***indicates p<0.001. Each data point represents the mean ± S.E. analyzed by *ANOVA*.

The fluorescent Ca^2+^ indicator Fura-2 was used to measure [Ca^2+^]_i_ in electrically stimulated (1 Hz, 5–10 volts) cardiac myocytes that had been freshly isolated from adult mice. We found that H_2_O_2_ (25 µM) promotes an increase in [Ca^2+^]_i_, measured as the ratio of F_340_/F_380_ (Figure 1A). We next treated cardiac myocytes with nifedipine, an extensively characterized L-type Ca^2+^ channel-blocking drug, to probe the role of L-type Ca^2+^ channels in the H_2_O_2_–stimulated responses observed in these cells. As shown in Figure 1A, the H_2_O_2_-promoted increase in cell-derived Fura-2 fluorescence is blocked by nifedipine. Similarly, the H_2_O_2_-promoted increase in cardiac myocyte contractility is abrogated by pre-treatment of the cells with nifedipine (Figure 1B). We also performed experiments comparing H_2_O_2_- and isoproterenol-promoted changes both in [Ca^2+^]_i_ and cardiac myocyte contractility. As shown in Figures 1C and 1D, the magnitude of both the H_2_O_2_-promoted contractility and Ca^2+^ responses are ∼70% of the responses seen following treatment with the β-adrenergic agonist isoproterenol.

**Figure 2:**
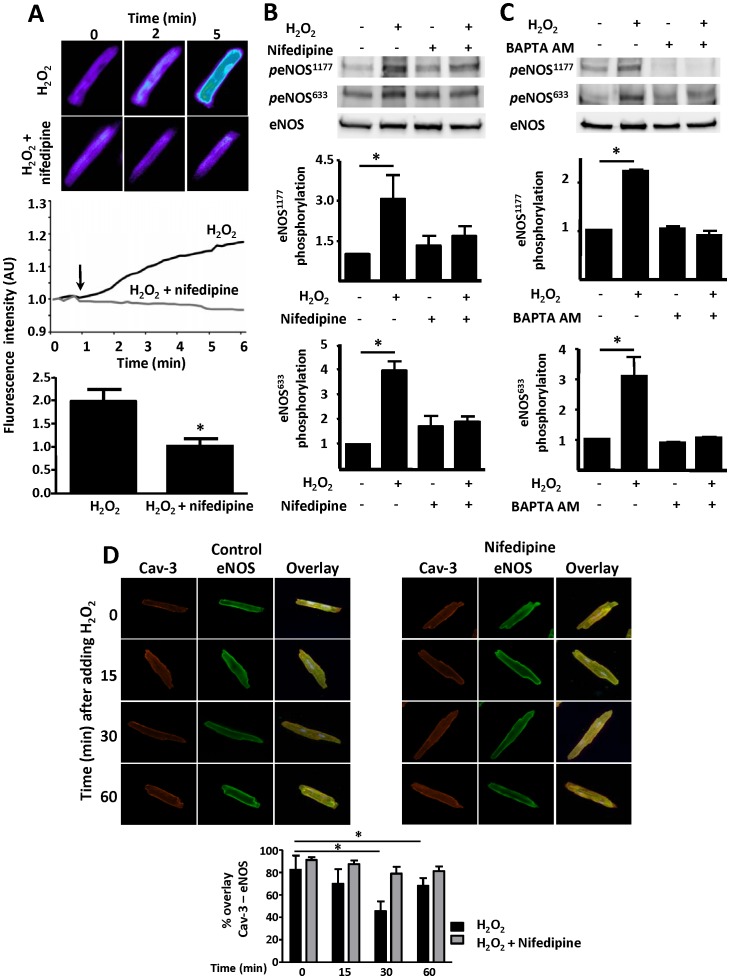
Nifedipine effects on H_2_O_2_-promoted NO synthesis, eNOS phosphorylation, and eNOS translocation. In Panel A, mouse cardiac myocytes were loaded with the NO chemical sensor Cu_2_(FL2E), and then treated with nifedipine (100 µM) or vehicle followed by hydrogen peroxide (H_2_O_2_, 10 µM) treatment. Upper panel shows representative fluorescence images at 0, 2, and 5 minutes followed treatments as indicated. Middle panel shows representative fluorescence tracings of single cells treated with H_2_O_2_ or H_2_O_2_ in the presence of nifedipine. Lower panel shows the results of pooled data analyzed from at least three independent repetitions with a minimum of 4 cells analyzed per experiment that yielded equivalent results; *indicates p<0.05. In Panel B, cardiac myocytes were incubated with nifedipine (100 µM, 30 min) or vehicle, then treated with hydrogen peroxide (H_2_O_2_, 25 µM, 15 min) and analyzed in immunoblots probed with antibodies as shown. Panel C shows immunoblot analyses from cardiac myocytes incubated with the intracellular calcium chelator BAPTA AM (60 µM, 30 min) or vehicle, then treated with H_2_O_2_ (25 µM, 15 min). Below each representative immunoblot the results of densitometric analyses from pooled data are shown, documenting the changes in phospho-eNOS1177 and phospho-eNOS633 plotted relative to the signals present in unstimulated cells. Each data point represents the mean ± S.E. derived from at least three independent experiments; *indicates p<0.05 (*ANOVA*). Panel D shows confocal microscopic images of cardiac myocytes treated with nifedipine (100 µM, 30 min) or vehicle, then treated with H_2_O_2_ (10 µM) for the indicated times. The cells were fixed, permeabilized, and probed with antibodies against total caveolin-3 (Alexa Fluor-Red 568) or eNOS (Alexa Fluor-Green 488); overlapping signals are shown in yellow. The bar graph below shows pooled data from three experiments, quantitating the percent overlap between eNOS and caveolin-3 at different times after adding H_2_O_2_. *indicates p<0.05 compared to t = 0.

Figure 2 presents the results of experiments using the NO chemical sensor Cu_2_(FL2E), which we previously used to explore the agonist-modulated regulation of cardiac myocyte NO synthesis [2]. H_2_O_2_-promoted NO synthesis is completely blocked by pre-treatment of the cells with nifedipine (100 µM, 30 min; Figure 2A). Nifedipine also abrogates the H_2_O_2_-promoted increase in eNOS phosphorylation (Figure 2B). The intracellular Ca^2+^ chelator BAPTA-AM blocks the H_2_O_2_-promoted increase in eNOS phosphorylation (Figure 2C). We previously demonstrated that H_2_O_2_ treatment of cardiac myocytes promotes reversible eNOS translocation from peripheral to internal membranes and back [2]. Figure 2D shows that H_2_O_2_-promoted eNOS translocation is completely blocked by nifedipine, without affecting the localization of the scaffolding/regulatory protein caveolin-3. Under these conditions, there is no change in total eNOS protein abundance in these cells, nor is there any apoptosis or necrosis of these cells (Figure S1A, F, and D).

**Figure 3:**
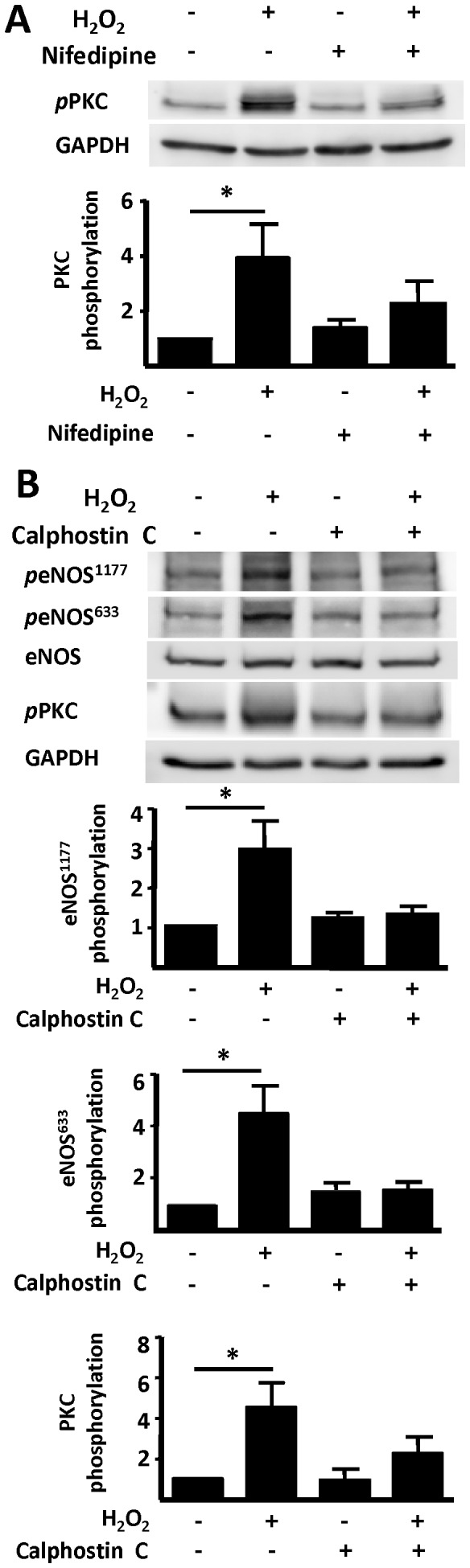
Effects of calphostin C on H_2_O_2_-promoted eNOS phosphorylation. In panel A, cardiac myocytes were incubated with nifedipine (100 µM, 30 min) or vehicle, then treated with H_2_O_2_ (25 µM) and analyzed in immunoblots probed with phospho-protein kinase C (PKC) phosphorylation (βII Ser660) or GAPDH antibodies. Panel B shows a representative experiment looking at the effects of calphostin C on H_2_O_2_-promoted eNOS phosphorylation. Freshly isolated adult murine cardiac myocytes were treated with calphostin C (1 µM, 30 min) or vehicle before treatment with H_2_O_2_ (25 µM, 15 min). Cell lysates were resolved by SDS-PAGE and probed using antibodies directed against phospho-eNOS Ser1177, phospho-eNOS Ser633, total eNOS, phospho-PKC, or GAPDH. Densitometric analyses from pooled data, plotting the fold increase of the degree of protein phosphorylation (in arbitrary units) relative to the signals present in unstimulated cardiac myocytes are also shown in this figure. Each data point represents the mean ± S.E. derived from three independent experiments, *indicates p<0.05 for respective phospho-protein versus unstimulated cells (*ANOVA*).

We next investigated the H_2_O_2_–stimulated Ca^2+^–modulated phosphorylation pathways regulating eNOS responses in these cells. As shown in Figure 3A, nifedipine blocks the H_2_O_2_-promoted increase in PKC phosphorylation. Because adult mouse cardiac myocytes are not amenable to RNA interference approaches, we used a series of protein kinase inhibitors to probe the pathways connecting H_2_O_2_ with eNOS phosphorylation. We found that the PKC inhibitor calphostin C blocks the increase in eNOS and PKC phosphorylations promoted by H_2_O_2_ (Figure 3B). We selected for analysis of eNOS phosphorylation the major band at M_r_ 135 kDa, which is the same M_r_ as the band seen in the total eNOS immunoblot. For PKC, the multiple bands seen may reflect the fact that we are using a “pan-phospho-PKC” antibody that picks up several different phospho-PKC isoforms; quantification of phospho-PKC includes all bands migrating in the vicinity of known PKC isoforms.

**Figure 4:**
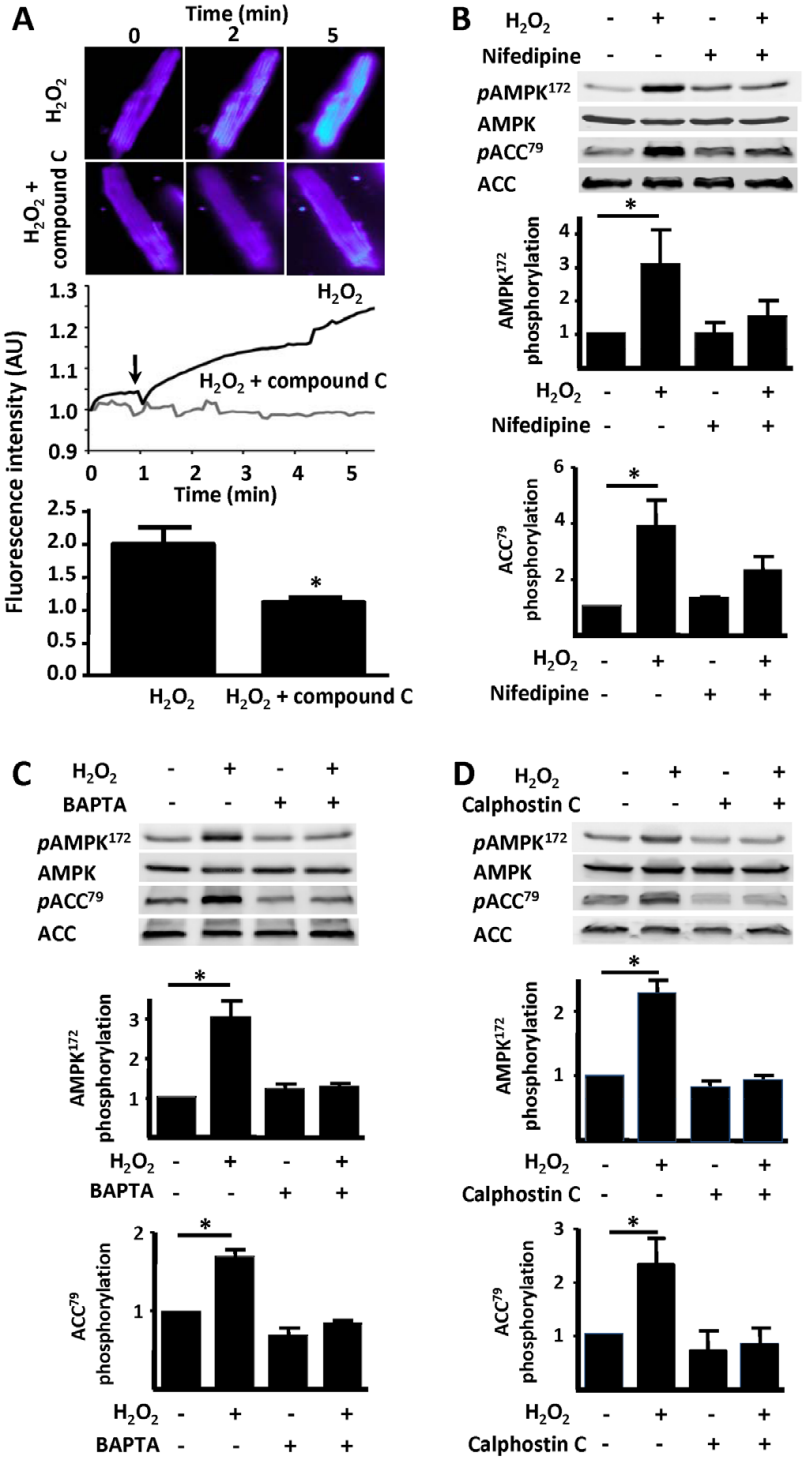
Intersections of kinase pathways and [Ca^2+^]_i_ in control of H_2_O_2_-promoted eNOS responses. In panel A, adult mouse cardiac myocytes were loaded with the NO dye Cu_2_(FL2E), and then treated with the AMPK inhibitor Compound C (1 µM) or vehicle followed by hydrogen peroxide (H_2_O_2_, 10 µM) treatment. Upper panel shows representative fluorescence images at 0, 2, and 5 minutes followed treatments as indicated. Middle panel shows representative fluorescence tracings of a cell treated with H_2_O_2_ or H_2_O_2_ in the presence of compound C. Lower panel shows the results of pooled data analyzed from at least three independent repetitions with a minimum of 4 cells analyzed per experiment that yielded equivalent results; *indicates p<0.05. Panels B, C, and D show representative experiments analyzing the effects of nifedipine (100 µM), BAPTA AM, (60 µM), or calphostin C (1 µM), on H_2_O_2_-promoted AMPK or ACC phosphorylation. Cardiac myocytes were pre-incubated with these compounds for 30 min, then treated with H_2_O_2_ (25 µM, 30 min) and analyzed in immunoblots probed with phospho-AMPK Thr172, phospho-ACC Ser79, AMPK, or ACC antibodies, as shown. Each data point represents the mean ± S.E. derived from at least three independent experiments; *indicates p<0.05 (*ANOVA*).

**Figure 5:**
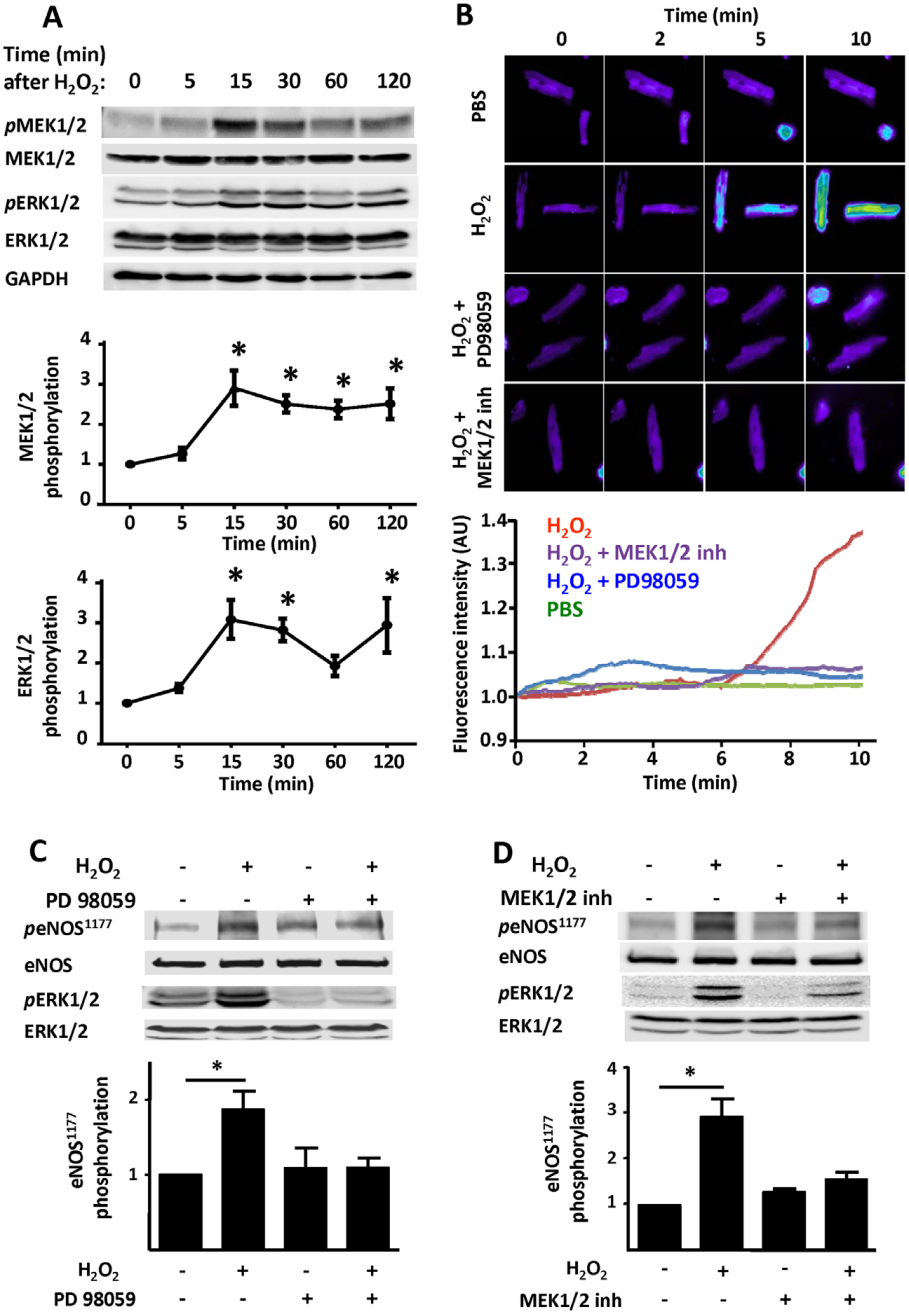
Effects of MAP kinase inhibitors on H_2_O_2_-promoted eNOS activation and phosphorylation. Panel A shows the results of immunoblots analyzed in lysates prepared from adult murine cardiac myocytes treated with hydrogen peroxide (H_2_O_2_, 25 µM) for the indicated times. Cell lysates were analyzed in immunoblots probed using antibodies directed against phospho-MEK (Ser217/221), phospho-ERK1/2 (Thr202/Tyr204), total MEK, ERK, and GAPDH, as indicated. Below each immunoblot are the results of densitometric analyses from pooled data, showing the fold increase in protein phosphorylations (in arbitrary units) in cardiac myocytes treated with H_2_O_2_ at the indicated times plotted relative to the signals present in unstimulated cells. Each data point represents the mean ± SE derived from three independent experiments. The results are significant at the p<0.05 level. *indicates p<0.05 (*ANOVA*). Panel B adult mouse cardiac myocytes were loaded with the NO dye Cu_2_(FL2E), and then treated with PD98059 (37.4 µM), MEK inhibitor (1 µM) or vehicle followed by hydrogen peroxide (H_2_O_2_, 10 µM) treatment. Upper panel shows representative fluorescence images at 0, 2, 5, and 10 minutes followed treatments as indicated. Lower panel shows representative fluorescence tracings of a cell treated with PBS (green line), H_2_O_2_ (red line), H_2_O_2_ in the presence of MEK1/2 inhibitor (purple line), or H_2_O_2_ in the presence of PD98059 (blue line). The results shown are representative of three independent experiments that yielded equivalent results. In panel C, cardiac myocytes were incubated with PD98059 (50 µM, 30 min) or vehicle, then treated with H_2_O_2_ (25 µM, 15 min) and analyzed in immunoblots probed with antibodies as shown. Panel D shows immunoblot analyses from cardiac myocytes incubated with MEK inhibitor (1 µM, 30 min) or vehicle, then treated with H_2_O_2_ (25 µM, 15 min). Below each representative immunoblot are shown the results of densitometric analyses from pooled data, documenting the changes in phospho-eNOS (Ser1177) plotted relative to the signal present in unstimulated cells. Each data point represents the mean ± S.E. derived from at least three independent experiments; *indicates p<0.05 (*ANOVA*).

**Figure 6:**
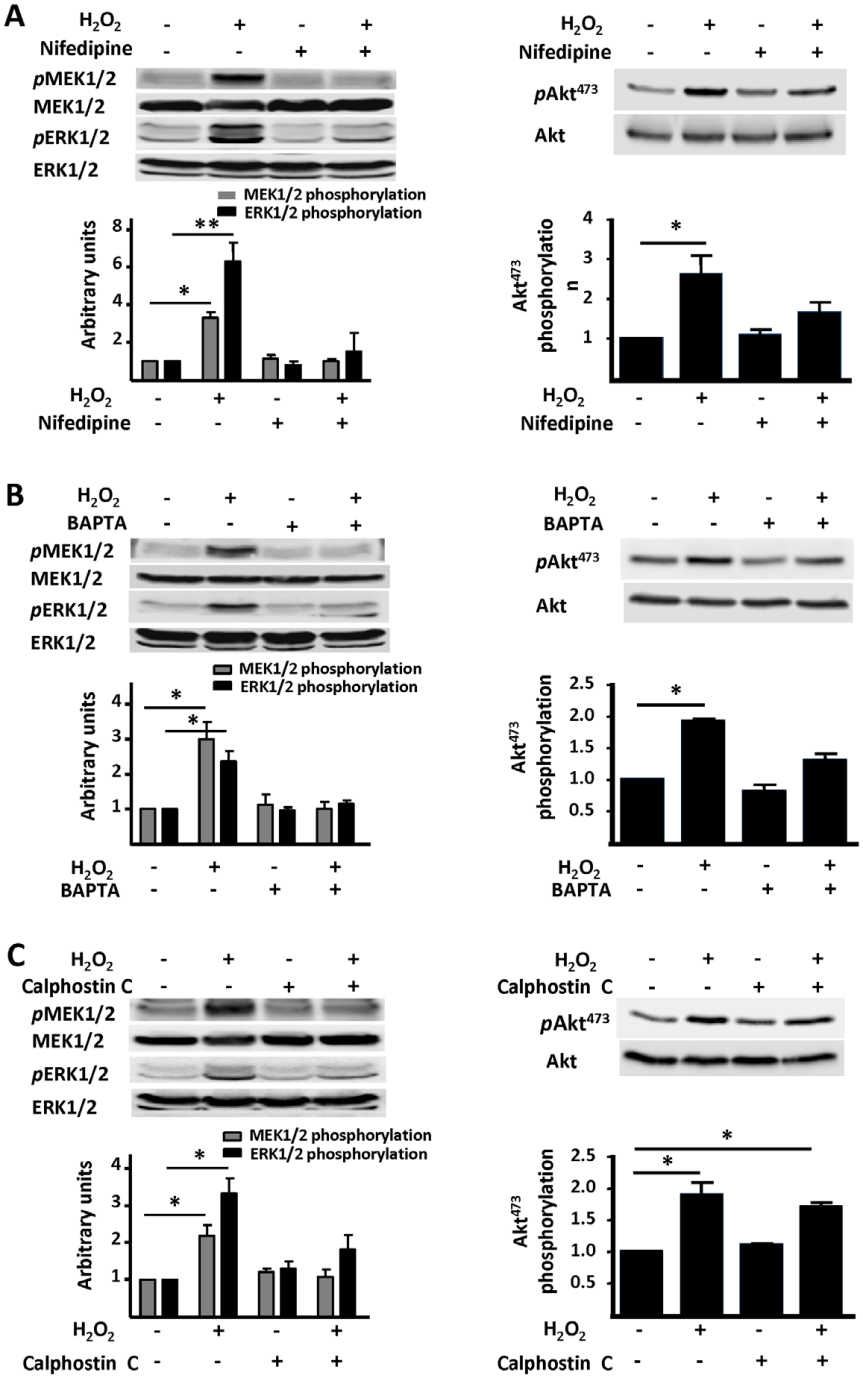
Pathways controlling H_2_O_2_-promoted phosphorylation of MEK/ERK 1/2 and Akt. In panel A, cardiac myocytes were incubated with nifedipine (100 µM, 30 min) or vehicle, then treated with hydrogen peroxide (H_2_O_2_, 25 µM, 15 min) and analyzed in immunoblots probed with antibodies as shown. Panel B shows immunoblot analyses from cardiac myocytes incubated with BAPTA AM (60 µM, 30 min) or vehicle, then treated with H_2_O_2_. Panel C shows cardiac myocytes treated with calphostin C (1 µM, 30 min) prior treatment with H_2_O_2_. Below each representative immunoblot are shown the results of densitometric analyses from pooled data, documenting the changes in phospho-MEK1/2 (Ser217/221), phospho-ERK1/2 (Thr202/Tyr204) (left panels), and phospho-Akt Ser473 (right panels) plotted relative to the signal present in unstimulated cells. Each data point represents the mean ± S.E. derived from at least three independent experiments (n = 4 for nifedipine, 3 for BAPTA and 6 for Calphostin C); *indicates p<0.05; **indicates p<0.01 (*ANOVA*).

We previously found [2] that the H_2_O_2_ promoted increase in eNOS phosphorylation depends on the AMP-activated protein kinase (AMPK). Here we show that the AMPK inhibitor Compound C blocks H_2_O_2_-induced cardiac myocyte NO synthesis, measured with the NO chemical sensor Cu_2_(FL2E) (Figure 4A). In order to investigate the role of L-type Ca^2+^ channel on the H_2_O_2_-promoted increase in AMPK phosphorylation, we analyzed immunoblots performed in cardiac myocyte lysates prepared from cells incubated with nifedipine (100 µM, 30 min) prior to H_2_O_2_ treatment (25 µM, 15 min) (Figure 4B). Nifedipine abrogates both the increase in AMPK phosphorylation as well as phosphorylation of the well-known AMPK substrate protein, acetyl-CoA carboxylase (ACC). The intracellular Ca^2+^ chelator BAPTA-AM also blocks H_2_O_2_–promoted AMPK and ACC phosphorylation (Figure 4C). Importantly, the PKC inhibitor calphostin C blocks H_2_O_2_–stimulated phosphorylation of AMPK and ACC (Figure 4D). H_2_O_2_ also promotes phosphorylation of the protein kinases MEK1/2 and ERK1/2 (Figure 5A) and of kinase Akt [2]. Inhibitors of MEK, including the structurally distinct kinase inhibitors PD98059 and “MEK1/2 inhibitor” block H_2_O_2_–stimulated NO synthesis (Figure 5B), and also attenuate H_2_O_2_–promoted phosphorylations of eNOS and ERK1/2 (Figures 5C and 5D; Figure S1D and E). The MEK1/2 and ERK1/2 phosphorylation responses are abrogated by nifedipine, BAPTA, or calphostin C (Figure 6).

**Figure 7:**
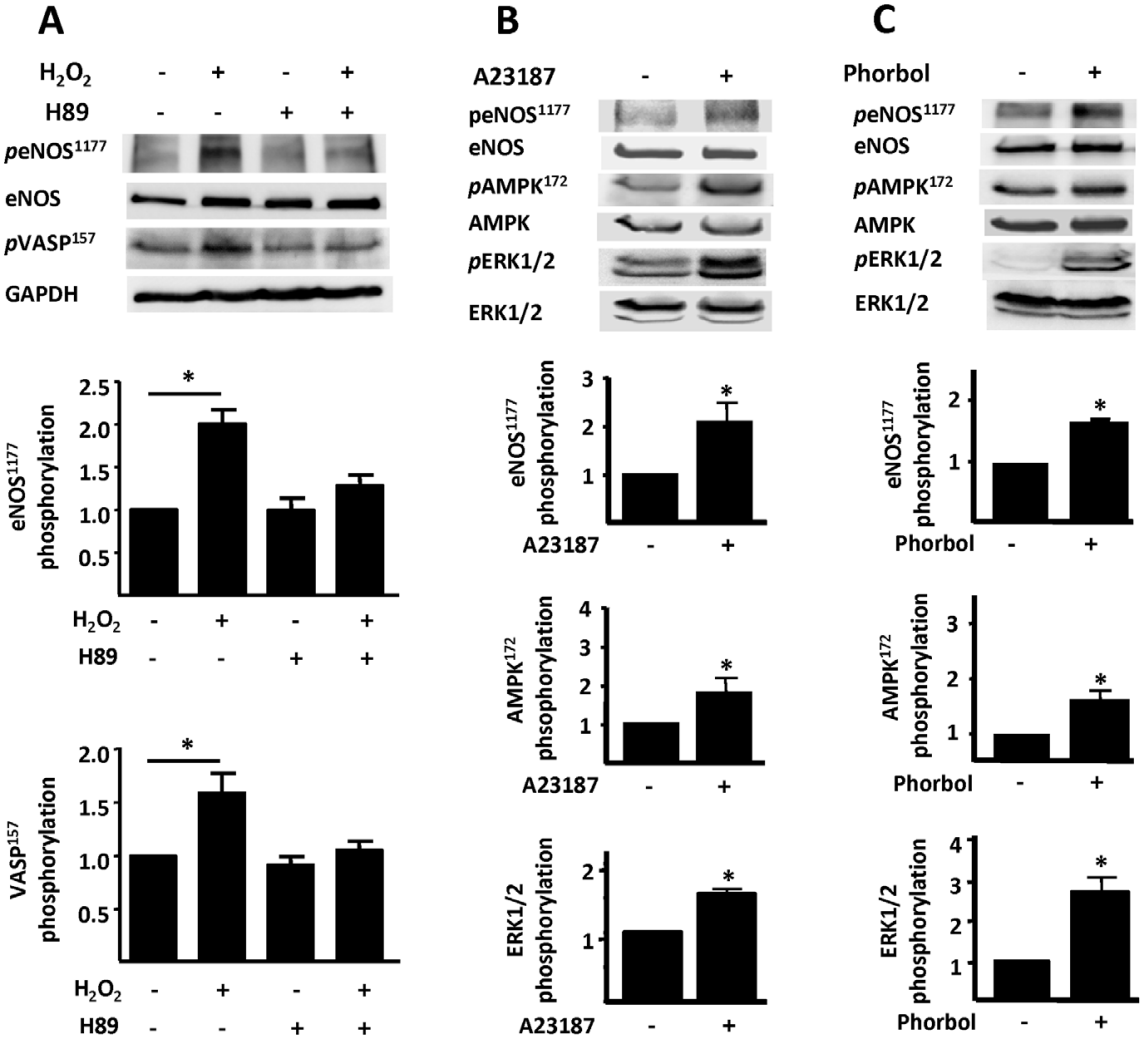
Effect of protein kinase A (PKA) inhibitor on H_2_O_2_-promoted eNOS phosphorylation. In panel A, cardiac myocytes were incubated with H89 (1 µM, 30 min) or vehicle, then treated with H_2_O_2_ (25 µM, 15 min) and analyzed in immunoblots probed with antibodies as shown. Below each representative immunoblot are shown the results of densitometric analyses from pooled data, documenting the changes in phospho-eNOS (Ser1177), and phospho-VASP (Ser157) plotted relative to the signals present in unstimulated cells. Each data point represents the mean ± S.E. derived from at least three independent experiments; *indicates p<0.05 (*ANOVA*). Panel B shows representative immunoblots from experiments documenting the effects of A23187 (40 µM, 5 min) on cardiac myocyte protein phosphorylation responses. Panel C shows the results of immunoblots analyzed in lysates prepared from cells treated with phorbol 12-myristate 13-acetate (10 µM, 15 min). Cell lysates were analyzed in immunoblots probed with antibodies as indicated. The immunoblot images shown are representative of three independent experiments that yielded similar results. Below each immunoblot panel are the results of densitometric analyses from pooled data, showing the fold increase in protein phosphorylation (in arbitrary units), *indicates p<0.05.

The phosphorylation response of kinase Akt to H_2_O_2_ appears to be differentially regulated: while H_2_O_2_–promoted Akt phosphorylation is blocked by nifedipine and BAPTA (as found for eNOS, AMPK, ERK1/2, and MEK1/2), calphostin C fails to attenuate Akt phosphorylation (Figure 6C). In contrast, the H_2_O_2_–stimulated phosphorylation of these other kinases is blocked by calphostin C (Figures 3B, 4D, and 6C). Moreover, H_2_O_2_–stimulated AMPK and Akt phosphorylations are unaffected by MAP kinase pathway inhibitors (Figure S1B and C). We next explored the role of PKA by investigating the effects of H_2_O_2_ on the phosphorylation of the protein VASP [1]. We probed immunoblots with phosphospecific antibodies directed against VASP phosphoserine 157, the preferred site for PKA-catalyzed VASP phosphorylation. As can be seen in Figure 7A, H_2_O_2_ promotes VASP phosphorylation, and the PKA inhibitor H89 completely blocks this phosphorylation response. Importantly, H_2_O_2_-promoted eNOS phosphorylation at ser1177 is blocked by this same PKA inhibitor (Figure 7A). Both calcium ionophore A23187 and the PKC agonist phorbol 12-myristate 13-acetate promote phosphorylation responses in these cells (Figure 7B and C).

## Discussion

These studies have used a combination of cellular imaging and biochemical approaches to explore eNOS activation and phosphorylation pathways in isolated mouse cardiac myocytes treated with H_2_O_2_. Several previous reports on the effects of ROS in cardiac myocytes have studied higher H_2_O_2_ concentrations and more prolonged treatments, which can lead to oxidative stress, Ca^2+^ overload, and myocyte apoptosis or necrosis [7], [8]. However, it is unlikely that the short-term exposure to low concentrations of H_2_O_2_ used in the present study cause cardiac myocyte membrane damage (Figure S1F); instead, our findings suggest a physiological role for H_2_O_2_ in the modulation of myocyte L-type Ca^2+^ channels. We found that H_2_O_2_-promoted increases in eNOS phosphorylation, NO production, and changes in eNOS subcellular localization in cardiac myocytes require L-type Ca^2+^ channel activity. Several lines of evidence in this study implicate Ca^2+^- and PKC-dependent signaling pathways as upstream determinants of H_2_O_2_-modulated responses in cardiac myocytes. We found that H_2_O_2_ treatment leads to increases in [Ca^2+^]_i_ in electrically stimulated cardiac myocytes, associated with an increase in myocyte contractility (Figure 1). These findings are in agreement with previous reports [9], [10]. Recent studies looking at H_2_O_2_-modulated calcium metabolism in cardiac myocytes have identified SERCA and NCX as important targets for H_2_O_2_ in these cells [11]. Our studies provide strong evidence for the involvement of L-type Ca^2+^ channels in modulating cardiac myocyte responses to H_2_O_2_.

Using the highly sensitive fluorescent probe Cu_2_(FL2E) to visualize NO synthesis in cardiac myocytes, we demonstrated that the L-type Ca^2+^ channel activity is required for H_2_O_2_-promoted NO synthesis (Figure 2A). We have previously shown that H_2_O_2_ activates the endothelial isoform of NOS in cardiac myocytes [2]. eNOS is a phosphoprotein that undergoes phosphorylation on multiple residues [12]. Here, we found that the increase in eNOS phosphorylation at serine 1177 and 633 residues caused by H_2_O_2_ exposure of cardiac myocytes was blocked by nifedipine or BAPTA-AM (Figure 2B and 2C). Because eNOS undergoes intracellular translocation following H_2_O_2_ treatment [2], we investigated the role of L-type Ca^2+^ channel on H_2_O_2_-promoted changes in eNOS intracellular localization. Caveolin-3 is a marker for the microdomains known as plasmalemmal caveolae. In cardiac myocytes caveolin-3 is also a binding partner of eNOS [13], [14]. As shown in Figure 2D, the colocalization between eNOS and caveolin-3 decreases 15 to 30 minutes after the addition of H_2_O_2_; eNOS returns to peripheral membranes and starts to re-localize with caveolin-3 ∼60 minutes after the addition of H_2_O_2_. Importantly, nifedipine abrogates H_2_O_2_-promoted eNOS translocation. There is no change in eNOS abundance or cardiac myocyte viability following treatment with H_2_O_2_ under these conditions (Figure S1A). Taken all together, these findings reveal that the H_2_O_2_-promoted increases in NO synthesis and eNOS phosphorylation depend on L-type Ca^2+^ channel activity and are associated with dynamic eNOS translocation.

Several protein kinases phosphorylate eNOS [12], including PKC, which stimulates NO production in endothelial cells associated with increased eNOS phosphorylation [15], [16]. In cultured cardiac myocytes, PKC isoforms regulate contractility and hypertrophy [17]. Activation of classical PKC isoforms is modulated by Ca^2+^ and diacylglycerol [18], [19]. We found that exposure to H_2_O_2_ leads to an increase in PKC phosphorylation, and confirmed that blockade of the L-type Ca^2+^ channel by nifedipine abrogates the phosphorylation response (Figure 3A). Inhibition of PKC using calphostin C blocked H_2_O_2_-promoted increase in eNOS phosphorylation (Figure 3B). These lines of evidence point to a central role for Ca^2+^- and PKC-dependent pathways in modulating H_2_O_2_-mediated eNOS activation, and are consistent with our finding that H_2_O_2_-dependent NO synthesis is blocked in cardiac myocytes treated with nifedipine.

**Figure 8:**
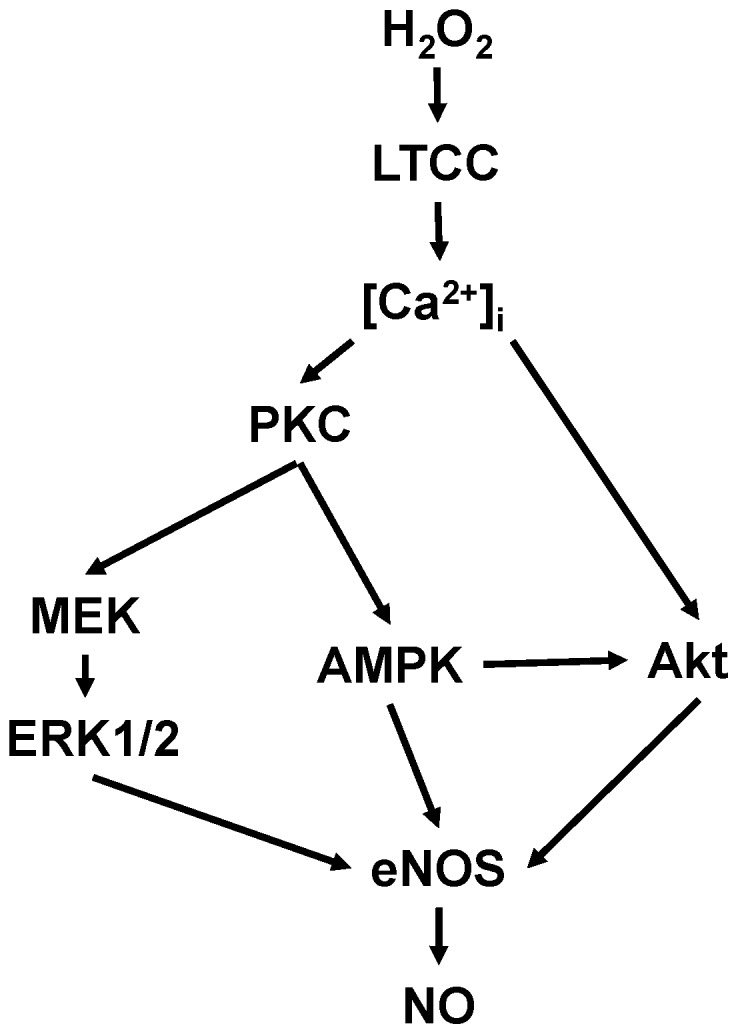
Scheme for H_2_O_2_-mediated regulation of eNOS signaling in cardiac myocytes. In this model, H_2_O_2_ activates the L-type Ca^2+^ channel (LTCC), causing an elevation in [Ca^2+^]_i_. The increase in [Ca^2+^]_i_ promotes phosphorylation and activation of protein kinases PKC and Akt, which lead to an increase in eNOS phosphorylation. Activation of PKC is required for the phosphorylation of MEK1/2, ERK1/2, and AMPK, which in turn promote eNOS phosphorylation and an increase in NO synthesis. See the text for additional discussion.

The AMP-activated protein kinase (AMPK) is a serine/threonine protein kinase that has been characterized as a sensor of cellular energy balance in mammalian cells [20]. We and others have previously reported that AMPK regulates eNOS in endothelial cells [21], [22]. Using the NO sensor Cu_2_(FL2E), we demonstrate here that activation of AMPK is required for the H_2_O_2_-promoted increase in cardiac myocyte NO synthesis (Figure 4A). Similar to eNOS, H_2_O_2_-promoted AMPK activation is Ca^2+^ and PKC dependent (Figure 4B, 4C and 4D). Nifedipine treatment of cardiac myocytes not only abrogates the increase in AMPK phosphorylation but also blocks phosphorylation of its substrate ACC (Figure 4B). A23187 calcium ionophore and phorbol 12-myristate 13-acetate treatments of cardiac myocytes enhanced AMPK phosphorylation (Figures 7B and 7C). These findings are consistent with previous observations in other experimental systems suggesting that AMPK can be activated by Ca^2+^/calmodulin [23]. In addition to AMPK, MEK1/2 appears to be necessary for the H_2_O_2_-promoted increase in cardiac myocyte NO synthesis and eNOS phosphorylation (Figure 5). Although both AMPK and kinase Akt are known to directly phosphorylate eNOS, the mechanisms whereby MEK1/2 and ERK1/2 modulate eNOS phosphorylation and activation are less clearly understood. Clearly, the modulation of cardiac myocyte eNOS by H_2_O_2_ involves complex interactions implicating multiple protein kinase pathways (Figure 8).

We have previously shown that both the PI3-K inhibitor wortmannin and Akt inhibitor XI block H_2_O_2_-promoted eNOS phosphorylation, and we also found that these inhibitors do not attenuate H_2_O_2_-promoted AMPK phosphorylation [2]. On the other hand, inhibition of AMPK by compound C reduces the H_2_O_2_-promoted increase in Akt phosphorylation, suggesting that AMPK may lie upstream of Akt in cardiac myocytes, as previously shown in vascular endothelial cells [21]. The inhibition of H_2_O_2_-promoted eNOS phosphorylation by the PKA inhibitor H89 (Figure 7A) implicates a role for PKA in modulating the response to H_2_O_2_; this hypothesis is further supported by our finding that H_2_O_2_ promotes VASP phosphorylation at a serine residue that is preferentially targeted by PKA (Figure 7A). The current studies have also explored whether changes in [Ca^2+^]_i_ or PKC activity are involved in the H_2_O_2_-promoted increase in cardiac myocyte Akt phosphorylation. Nifedipine and BAPTA abrogate the H_2_O_2_-promoted increase in Akt phosphorylation (Figure 6A and B). These observations are consistent with previous reports in other cell systems, which suggested that PI3-K/Akt can be activated by intracellular Ca^2+^ fluxes in endothelial cells [24]. Importantly, the H_2_O_2_-promoted increase in Akt phosphorylation is unaffected by the PKC inhibitor calphostin C, indicating that signaling to Akt by H_2_O_2_ does not involve PKC activation (Figure 6C).

The present studies define a critical role for L-type Ca^2+^ channel activity in the control of H_2_O_2_–dependent pathways that lead to the phosphorylation of protein kinases regulating eNOS signaling in cardiac myocytes. The physiological effects of low H_2_O_2_ concentrations seen in these studies can be contrasted to the much higher levels of oxidative stress that have been observed in cardiac disease states, including heart failure and cardiomyopathy [25], [26], [27]. A deeper understanding of the factors that modulate H_2_O_2_ metabolism in cardiac myocytes is needed in order to devise therapeutic strategies to regulate ROS balance in physiological and pathophysiological states in the heart.

## Materials and Methods

### Materials

Polyclonal antibodies directed against phospho-eNOS (Ser1177), phospho-PKC (pan) (βII Ser660), phospho-AMPK (Thr172), phospho-Akt (Ser473), phospho-ACC (Ser79), phospho-MEK1/2 (Ser217/212), phospho-ERK1/2 (Thr202/Tyr204), AMPK, Akt, ACC, MEK1/2, ERK1/2, phospho-VASP (Ser157), and H-89 were from Cell Signaling Technologies (Beverly, MA). Antibodies against total eNOS, total VASP, Caveolin-3, and phospho-eNOS (Ser633) were from BD Transduction Laboratories (Lexington, KY). Collagenase type 2 was from Worthington Biochemical (Lakewood, NJ). Compound C, PD98059 (a selective cell-permeable inhibitor of MAP kinase kinase [MEK]), “MEK1/2 inhibitor” (a cell-permeable vinylogous cyanamide that acts as a selective inhibitor of MEK1/2), and Calphostin C were from Calbiochem. Super Signal substrate for chemiluminescence detection and secondary antibodies conjugated with horseradish peroxidase were from Pierce. Tris-buffered saline and phosphate-buffered saline were from Boston Bioproducts (Ashland, MA). Laminin was from BD Bioscience (San Jose, CA). Minimum essential medium with Hank’s balanced salt solution and glutamine were from Gibco-BRL. Calf serum was from HyClone (Logan, UT). The AlexaFluor488 Annexin V/Dead cell apoptosis kit, Alexa Fluor-Green (488)-tagged goat anti-rabbit antibody and Alexa Fluor red (568)-tagged goat anti-mouse antibody were from Invitrogen/Molecular Probes. All other reagents were from Sigma (Plymouth Meeting, PA). Mouse line C57BL6/J was from Jackson Labs (Bar Harbor, ME). Cu_2_(FL2E) was synthesized as previously reported [28].

### Isolation of Adult Mouse Ventricular Myocytes

All animal experimentation was performed according to protocols approved by the Harvard Medical School Committee on Use of Animals in Research. For these studies, 8–10-week-old C57BL6/J, mice were lightly anesthetized with isofluorane, heparinized (50 U, ip), and sacrificed. The heart was quickly removed from the chest and retrogradely perfused through the aorta as described [29]. Cardiac myocyte isolation methods followed the described procedures [29], with minor modifications as previously reported [1]. In brief, enzymatic digestion was initiated by adding collagenase type 2 to the cardiac perfusion solution, followed by the stepwise introduction of CaCl_2_, after which the heart tissue was minced and the cells were dispersed by trituration. Subsequently, the cardiac myocytes were allowed to settle, and then washed, pelleted, counted, and plated.

### Cell Culture

Cardiac myocytes were plated in laminin-coated 6-well culture dishes (50,000 rod-shaped cells per dish) in plating medium consisting of Minimum Essential Medium with Hank’s balanced salt solution, supplemented with calf serum (10% v/v), 2,3-butanedione monoxime (10 mM), penicillin-streptomycin (100 U/ml), glutamine (2 mM), and ATP (2 mM). After the cells were attached (∼1 hour), the plating medium was changed to culture medium consisting of Minimum Essential Medium with Hank’s balanced salt solution, supplemented with bovine serum albumin (1 mg/ml), penicillin-streptomycin (100 U/ml), and glutamine (2 mM) and the cells were cultured for 4 hours.

### Measurements of Intracellular Ca^2+^ by Fura-2

Intracellular calcium concentrations were monitored using electrically stimulated freshly isolated cardiac myocytes. In brief, coverslips of cardiac myocytes loaded with Fura-2AM (1 µM, 20 min, room temperature) were used to monitor intracellular calcium transients. Fura-2 fluorescence was measured using an IonOptix spectrophotometer (HyperSwitch; IonOptix, Milton, MA, USA). Fura-2 was excited by light at 340-nm and 380-nm. A photomultiplier tube detected the emitted fluorescence at 510 nm. Experiments were performed at room temperature on the stage of an inverted microscope (Nikon, Tokyo, Japan), and myocytes were visualized using an air objective (S Fluor 40X). Field stimulation (5–10 V, 1 Hz) was accomplished using the MyoPacer (IonOptix). In all experiments, myocytes were kept in Tyrode’s solution (pH 7.45 with 1.0 mM CaCl_2_ added). A two-point Fura2 calibration was performed according to the method of Grynkiewicz et al. [30], [31].

### Myocyte Sarcomere Shortening

Myocytes were placed in a rapid change stimulation chamber on an inverted Nikon microscope stage and continuously bathed in Tyrode's solution at room temperature, pH 7.45 with 1.0 mM CaCl_2_ added. Myocytes were field-stimulated (MyoPacer Field Stimulator, IonOptix, Milton, MA) at 1 Hz, 5–10 Volts. Sarcomere length was recorded with a video edge detector coupled to a camera (MyoCam-S, IonOptix). Sarcomere shortening analyses was performed using IonWizard Core Analysis software (IonOptix) in myocytes without any treatment and after 5 to 10 min of H_2_O_2_. In some studies, myocytes were pre-treated with nifedipine (100 µM) for at least 15 min before and during H_2_O_2_ treatment. Sarcomere shortening was expressed as percent shortening relative to the resting diastolic length.

### Intracellular Nitric Oxide Imaging

Cardiac myocytes harvested from at least three independent preparations were analyzed. The signal from the NO sensor was analyzed as the slope of the fluorescence increase observed following the addition of agonist or vehicle. Cells were cultured on cover slips and loaded with 5 µM Cu_2_(FL2E) [28] for 2 hours in Tyrode’s solution at 37°C and 2% CO_2_. Cover slips were then placed in an onstage incubator (Tokai, Tokyo, Japan) on an Olympus IX81 inverted microscope equipped with an UPlan 40X/1.3 oil objective in a low-volume glass-covered recording chamber. Fluorescence signals were analyzed by using a Hamamatsu Orca CCD camera (Hamamatsu, Tokyo, Japan) at 470 nm. Viable rod-shaped cardiac myocytes with rectangular ends were selected by differential interface contrast imaging and then subjected to fluorescence imaging, following treatments as indicated.

### Immunoblot Analyses

After drug treatments, cardiac myocytes were washed with PBS buffer and incubated for 10 minutes in lysis buffer (50 mM Tris-HCl, pH 7.4; 150 mM NaCl; 1% Nonidet P-40; 0.25% sodium deoxycholate; 1 mM EDTA; 2 mM Na_3_VO_4_; 1 mM NaF; 2 µg/mL leupeptin; 2 µg/ml antipain; 2 µg/ml soybean trypsin inhibitor; and 2 µg/mL lima trypsin inhibitor). Cells were harvested by scraping. After separation by SDS-PAGE, proteins were electroblotted onto nitrocellulose membranes. After incubating the membranes in 5% nonfat dry milk in Tris-buffered saline with 0.1% (vol/vol) Tween 20 (TBST), membranes were incubated overnight in TBST containing 5% bovine serum albumin plus the specified primary antibody. After four washes (10 min each) with TBST, the membranes were incubated for one hour with a horseradish peroxidase-labeled goat anti-rabbit or anti-mouse immunoglobulin secondary antibody in TBST containing 1% milk. The membranes were washed four additional times in TBST, then incubated with a chemiluminescent reagent according to the manufacturer's protocols (SuperSignal West Femto), and digitally imaged in a chemiluminescence imaging system (Alpha Innotech Corporation, San Leandro, CA). Quantitative analyses of the chemiluminescent signals were performed using an AlphaEase®FC software (Alpha Innotech, San Leandro, CA). For quantitative analyses of immunoblot experiments, the signal is normalized to the value obtained in the absence of added drug.

### Immunohistochemistry

Cardiac myocytes plated on 8-well-chamber slides (Thermo Scientific) were fixed in 4% paraformaldehyde for 20 min, rinsed twice with PBS, permeabilized in 0.1% Triton X-100 for 45 min, and blocked with 10% goat serum overnight. Immunoreactive eNOS and caveolin-3 were co-localized using confocal microscopy. After incubating with both primary antibodies (in blocking solution at 4°C, overnight), samples were washed three times in PBS for 10 min. The eNOS primary antibody was localized by immunofluorescent detection with a secondary Alexa Fluor-Green (488)-tagged goat anti-rabbit antibody (1∶200 dilution, 1 h incubation), and Cav-3 primary antibody was detected with a secondary Alexa Fluor red (568)-tagged goat anti-mouse antibody (1∶200 dilution, 1 h incubation). Samples were washed three times in PBS for 10 min to remove excess secondary antibody and then mounted on slides using medium containing 4',6-diamidino-2-phenylindole as nuclear counter stain. Microscopic analysis of samples was performed using an Olympus IX81 inverted microscope in conjunction with a DSU spinning disk confocal system equipped with a Hamamatsu Orca ER cooled-CCD camera. Images were acquired using a 40X/1.3 differential interference contrast oil immersion objective lens and analyzed using Metamorph software from Universal Imaging, Inc. (Downingtown, PA).

### Measurement of Cell Viability and Apoptosis

Cardiac myocytes were plated on laminin-coated culture dishes in Tyrode's solution at room temperature, pH 7.45 with 1.0 mM CaCl_2_ added. Cardiac myocytes were treated with varying concentrations of H_2_O_2_ for 15 minutes. Cell viability was determined by the ratio of rod-shaped to total cells. Apoptosis and necrosis were detected using an AlexaFluor488 annexin V/propidium iodide detection kit (Invitrogen/Molecular Probes). Briefly, cardiac myocytes were incubated with annexin V and propidium iodide for 10 minutes at room temperature. Dishes were photographed under both phase-contrast and fluorescence microcopy, and rod-shaped (viable), rounded (non-viable), and total cells were counted. Apoptotic cardiac myocytes were defined as annexin V-positive (green-stained cells) and necrotic myocytes as annexin V plus propidium iodide-positive cells (green- and red-stained cells).

### Statistical Analysis

Mean values for individual experiments were expressed as means ± S.E. Statistical differences were assessed by *ANOVA*. A p value of less than 0.05 was considered significant.

## Supporting Information

Figure S1 In Panel A, cardiac myocytes were treated with hydrogen peroxide (H_2_O_2_, 25 µM) and analyzed in immunoblots probed with antibodies as shown. The immunoblots shown are representative of three independent experiments that yielded similar results. Panel B shows immunoblot analyses from cardiac myocytes incubated with PD98959 (50 µM, 30 min) or vehicle, then treated with H_2_O_2_ (25 µM, 15 min). Panel C shows representative immunoblot analyses from cells incubated with MEK1/2 inhibitor (1 µM, 30 min) or vehicle, then treated with H_2_O_2_. The immunoblots in panel B and C were probed with antibodies against phospho-Akt (Ser 473) or phospho-AMPK (Ser172). Panels D and E show results of pooled data corresponding to representative experiments shown in Figure 5 (panels C and D). In panel F, cardiac myocytes were treated with vehicle, H_2_O_2_ (25 µM), or H_2_O_2_ (500 µM) for 15 min and stained with annexin V and propidium iodide as described in the text. The two fluorescence channels were obtained sequentially; overlaying of the differential interference contrast image (DIC) and both fluorescence channels (annexin V and propidium iodide) is shown. Panel G shows the percentage of apoptotic (annexin V positive) and necrotic (annexin V + propidium iodide positive) cardiac myocytes. Panel H shows the percentage of viable (rod-shaped) and nonviable (round) cardiac myocytes. *indicates p<0.05; **indicates p<0.01; and ***indicates p<0.001 (*ANOVA*).
(TIF)
