# Supplementary figures and images for: Role of Ca2+ in the Control of H2O2-Modulated Phosphorylation Pathways Leading to eNOS Activation in Cardiac Myocytes
Source: PLoS One. 2012 Sep 6;7(9):e44627. doi: 10.1371/journal.pone.0044627 (PMC3435284; doi:10.1371/journal.pone.0044627)

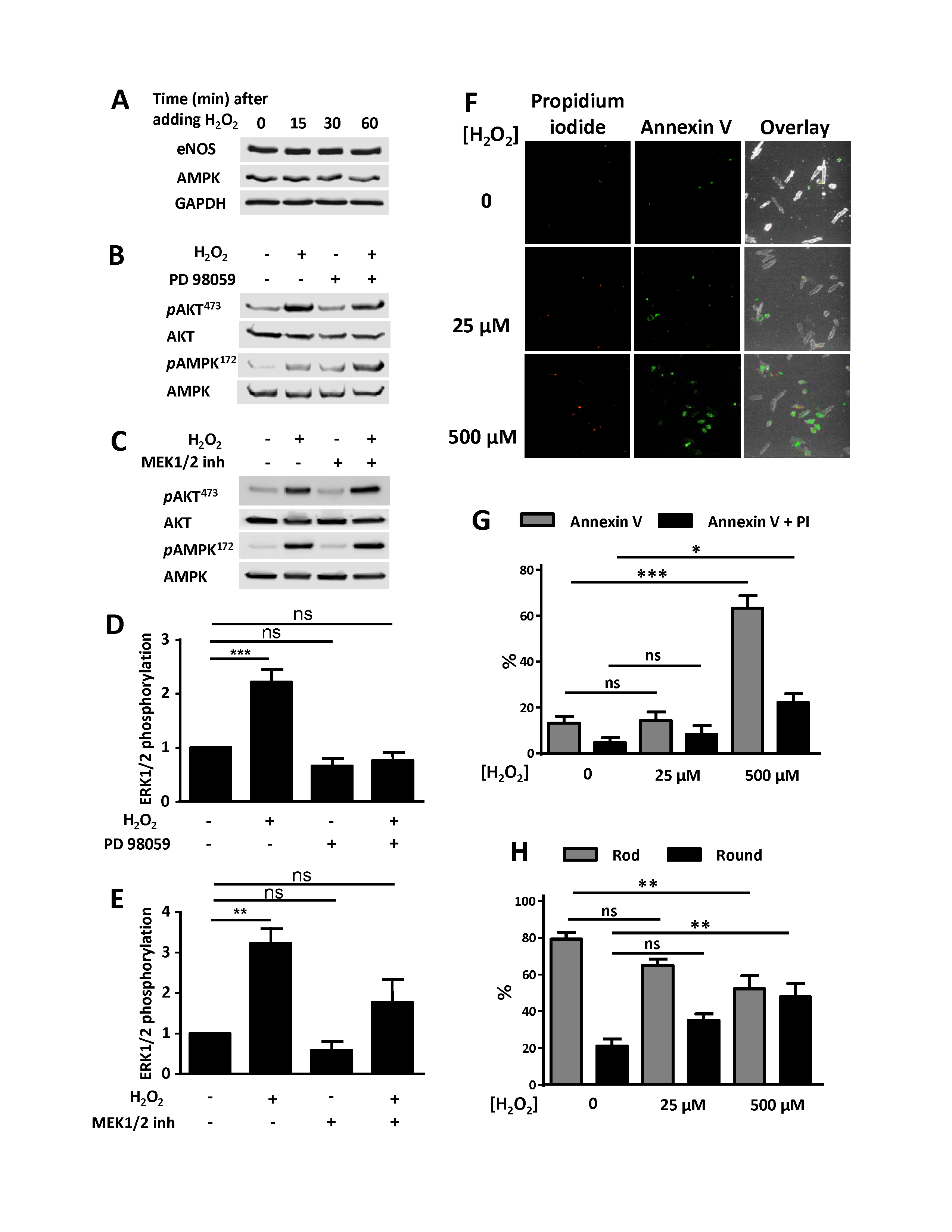

Supplement: Figure S1 — In Panel A, cardiac myocytes were treated with hydrogen peroxide (H2O2, 25 µM) and analyzed in immunoblots probed with antibodies as shown. The immunoblots shown are representative of three independent experiments that yielded similar results. Panel B shows immunoblot analyses from cardiac myocytes incubated with PD98959 (50 µM, 30 min) or vehicle, then treated with H2O2 (25 µM, 15 min). Panel C shows representative immunoblot analyses from cells incubated with MEK1/2 inhibitor (1 µM, 30 min) or vehicle, then treated with H2O2. The immunoblots in panel B and C were probed with antibodies against phospho-Akt (Ser 473) or phospho-AMPK (Ser172). Panels D and E show results of pooled data corresponding to representative experiments shown in Figure 5 (panels C and D). In panel F, cardiac myocytes were treated with vehicle, H2O2 (25 µM), or H2O2 (500 µM) for 15 min and stained with annexin V and propidium iodide as described in the text. The two fluorescence channels were obtained sequentially; overlaying of the differential interference contrast image (DIC) and both fluorescence channels (annexin V and propidium iodide) is shown. Panel G shows the percentage of apoptotic (annexin V positive) and necrotic (annexin V + propidium iodide positive) cardiac myocytes. Panel H shows the percentage of viable (rod-shaped) and nonviable (round) cardiac myocytes. *indicates p<0.05; **indicates p<0.01; and ***indicates p<0.001 (ANOVA). (TIF) [file pone.0044627.s001.tif]
